# Supplementary material for: Stacking Model‐Based Classifiers for Dealing With Multiple Sets of Noisy Labels
Source: Biom J. 2025 Mar 12;67(2):e70042. doi: 10.1002/bimj.70042 (PMC11898607; doi:10.1002/bimj.70042)
Supplement: Supplementary file 1 — Supporting Information [file BIMJ-67-e70042-s001.zip › density-based-ensemble-model-main/competitors_real_data/netlab-master/netlab-master/help/nethess.htm]

Netlab Reference Manual nethess


# nethess

## Purpose

Evaluate network Hessian

## Synopsis

```
h = neterr(w, net, x, t)
[h, varargout] = neterr(w, net, x, t, varargin)
```

## Description

`h = nethess(w, net, x, t)` takes a weight vector `w` and a network
data structure `net`, together with the matrix `x` of input
vectors and the matrix `t` of target vectors, and returns the
value of the Hessian evaluated at `w`.

`[e, varargout] = nethess(w, net, x, t, varargin)` also returns any additional
return values from the network Hessian function, and passes additional arguments
to that function.

## Example

In `evidence`, this function is called once to compute the
data contribution to the Hessian

```
[h, dh] = nethess(w, net, x, t, dh);
```

and again to update the Hessian for new values of the hyper-parameters

```
h = nethess(w, net, x, t, dh);
```

## See Also

`neterr`, `netgrad`, `netopt`

---

**Pages:**
Index


---

Copyright (c) Ian T Nabney (1996-9)
